# Microsatellite instability and mutations in BRAF and KRAS are significant predictors of disseminated disease in colon cancer

**DOI:** 10.1186/s12885-015-1144-x

**Published:** 2015-03-14

**Authors:** Helgi Birgisson, Karolina Edlund, Ulrik Wallin, Lars Påhlman, Hanna Göransson Kultima, Markus Mayrhofer, Patrick Micke, Anders Isaksson, Johan Botling, Bengt Glimelius, Magnus Sundström

**Affiliations:** 1 Department of Surgical Sciences, Colorectal Surgery, Uppsala University, 75185 Uppsala, Sweden; 2 Department of Immunology, Genetics and Pathology, Uppsala University, 75185 Uppsala, Sweden; 3 Science for Life Laboratory, Department of Medical Sciences, Uppsala University, 75185 Uppsala, Sweden; 4 Department of Radiology, Oncology and Radiation Science, Uppsala University, 75185 Uppsala, Sweden

**Keywords:** Colon cancer, MSI, BRAF, KRAS, PIK3CA, DNA copy number, Prognosis

## Abstract

**Background:**

Molecular alterations are well studied in colon cancer, however there is still need for an improved understanding of their prognostic impact. This study aims to characterize colon cancer with regard to KRAS, BRAF, and PIK3CA mutations, microsatellite instability (MSI), and average DNA copy number, in connection with tumour dissemination and recurrence in patients with colon cancer.

**Methods:**

Disease stage II-IV colon cancer patients (n = 121) were selected. KRAS, BRAF, and PIK3CA mutation status was assessed by pyrosequencing and MSI was determined by analysis of mononucleotide repeat markers. Genome-wide average DNA copy number and allelic imbalance was evaluated by SNP array analysis.

**Results:**

Patients with mutated KRAS were more likely to experience disease dissemination (OR 2.75; 95% CI 1.28-6.04), whereas the opposite was observed for patients with BRAF mutation (OR 0.34; 95% 0.14-0.81) or MSI (OR 0.24; 95% 0.09-0.64). Also in the subset of patients with stage II-III disease, both MSI (OR 0.29; 95% 0.10-0.86) and BRAF mutation (OR 0.32; 95% 0.16-0.91) were related to lower risk of distant recurrence. However, average DNA copy number and PIK3CA mutations were not associated with disease dissemination.

**Conclusions:**

The present study revealed that tumour dissemination is less likely to occur in colon cancer patients with MSI and BRAF mutation, whereas the presence of a KRAS mutation increases the likelihood of disseminated disease.

**Electronic supplementary material:**

The online version of this article (doi:10.1186/s12885-015-1144-x) contains supplementary material, which is available to authorized users.

## Background

Colorectal cancer (CRC) is the third most common cancer and the second most common cause of cancer-related death in Sweden [1]. Metastatic disease is present at diagnosis in 20-25% of patients and another 20-25% develops metastases in the course of the follow-up time. As local disease nowadays rarely is a cause of death in cancer of the colon and rectum [2], tumour cell dissemination may be considered a prerequisite for tumour death. To be able to improve survival by more appropriate treatment selection in primary disease, focus must therefore be on the identification of tumours with the capability to disseminate, whether clinically apparent at diagnosis (stage IV) or detected during follow-up after curative surgery (stages II and III).

The TNM (tumour-node-metastasis) classification based on radiologic and histopathological evaluation is currently the most reliable method for treatment selection and prognostic prediction in patients with CRC [3]. Patients curatively operated for stage II disease have around 15% risk of developing disease recurrence [4] if staged appropriately, operated according to modern principles and assessed with high quality pathology. Due to low risk of recurrence, these patients are regularly not given adjuvant chemotherapy, unless they are considered to be at “high risk” due to poor prognostic features such as T4, emergency operation or vascular invasion [5,6]. Patients with stage III disease have approximately a 40% risk to develop recurrent disease. Adjuvant therapy with 5-fluorouracil (5-FU)/leucovorin in patients with stage III disease reduces this risk by approximately 30%. If 5-FU/leucovorin is combined with oxaliplatin, the recurrence rate is further decreased with 15-20% [7]*.* Obviously, a subgroup of patients with stage III disease is given adjuvant chemotherapy with limited survival benefits. At the same time, there is an under-treatment of the subset of stage II patients that eventually develop recurrent disease.

CRC is heterogeneous with regard to molecular alterations and characterization of the molecular aetiology of sporadic CRC has identified different oncogenic pathways. The two major genomic instability pathways are the “traditional” chromosomal instability (CIN), or aneuploidy pathway, and the microsatellite instability (MSI) pathway [8-11]. These two pathways have been described as mutually exclusive, as the CIN tumours are microsatellite stable (MSS) [12]. CIN positive tumours constitute 65-70% of CRCs and have been associated with an aggressive clinical behaviour and distal location [10,13]. Tumours with CIN usually have large genomic aberrations that lead to higher average DNA copy number compared with MSI tumours [14]. Absolute DNA copy numbers can be assayed by SNP arrays and subsequent allele-specific analysis [15]. The MSI phenotype is the result of gene silencing of DNA mismatch repair (MMR) genes that cause accumulation of mutations in tumour suppressor genes and oncogenes. The MSI phenotype is therefore also referred to as the MMR deficient or mutator phenotype. CRC with MSI accounts for approximately 15% of sporadic CRCs and is characterized by a more proximal location, mucinous differentiation, near-diploid chromosome set and better prognosis compared to MMR proficient, frequently CIN positive, CRC [16-19]. Some CRC tumours also display epigenetic instability manifested as CpG island methylator phenotype (CIMP) or global DNA hypomethylation. CIMP-positive tumours are strongly associated with the MSI phenotype and the presence of BRAF mutations [20,21]. An additional CRC subtype comprises MSS CIN negative (diploid) tumours that also frequently are CIMP positive and BRAF mutated [12].

CRC tumourigenesis is also dependent on mutations in genes that deregulate intracellular signaling pathways, e.g. the EGFR mitogen-activated protein kinase (MAPK) and phosphatidylinositol 3-kinase (PI3K) pathways. Frequently mutated genes in these pathways are KRAS, BRAF and PIK3CA. Similar to CIN and MSI, these genes have been suggested as prognostic biomarkers, but although examined in many previous studies, the precise prognostic role of mutations in these genes remains unclear [22,23]. Based on the increased molecular knowledge of CRC, a classification of sporadic CRC into five different entities has been proposed [12]. However, the clinical value of these entities is still unclear and conflicting data exists among studies, probably a result of the heterogeneity of CRC resulting in overlap between the different pathways involved in CRC tumourigenesis.

In order to better understand tumour cell characteristics in primary colon cancers associated with tumour cell dissemination, and disease recurrence, the aim of this study was to characterize colon tumours, stratified by tumour stage and presence or development of metastatic disease, with regard to KRAS, BRAF, and PIK3CA mutations, MSI, and average DNA copy number.

## Methods

### Patient material and study design

Fresh frozen tumour material was available for molecular analysis from over 600 patients with primary colon and rectal cancer operated at the Uppsala University Hospital, Sweden, between 1987 and 2006, or at the Central District Hospital in Västerås, Sweden, between 2000 and 2003. From this population patients with stage II and III tumours, with and without recurrent disease, and patients with stage IV disease at diagnosis, were identified. To enable comparisons of tumours with and without metastatic capability, patients with synchronous metastases at diagnosis were considered equivalent to those with metastases appearing during the follow-up period, as both synchronous and metachronous metastases develop from the primary tumour and may indicate the presence of certain traits. The terms “non-disseminated” was used for patients with stage II and III tumours without recurrence and “disseminated” for stages II and III with recurrence together with stage IV.

Only colon cancers were selected as rectal cancers are often treated preoperatively with radiation and/or chemotherapy and rectal cancer can differ from colon cancer in the mutation profile. To ensure the high quality of the study population, only radiologically adequately staged patients and those operated abdominally according to either right-sided or left-sided hemicolectomy or sigmoidectomy were included. No preoperative therapy was allowed and the surgery was required to be radical (R0). Patients with stage II disease were only included if at least 10 lymph nodes were analyzed. Moreover, patients with stages II-III, with no disease recurrence were only included if the follow-up time was longer than 5 years.

Haematoxylin-eosin stained tissue sections were prepared from OCT-embedded fresh-frozen specimens using a cryostat and the CryoJane tape-transfer system (Instrumedics, Richmond, IL). The tumour tissue sections were examined by a trained pathologist to ensure that only representative samples containing more than 40% tumour cells were included.

Based on the above-mentioned criteria, tumour tissue from 121 patients was selected for analysis; 25 with disease stage II and 28 with stage III without disease recurrence; 15 with stage II and 27 with stage III with distant recurrence and 26 with stage IV disease. Totally 68 patients were therefore regarded as disseminated and 53 as non-disseminated. The stage II group with disease recurrence had to be limited to 15 cases as no more eligible patients could be identified; otherwise the aim was to include at least 25 patients in each group. Basic clinical and histopathological information of the selected cohort is given in Additional file 1: Table S1.

### DNA extraction

Genomic DNA was extracted from 5-10 frozen tissue sections (10 μm) using the QIAamp DNA Mini Kit (Qiagen GmbH, Hilden, Germany) according to the manufacturer’s recommendations. The purityand concentration of the extracted DNA was assessed using a NanoDrop instrument (Thermo Scientific, Wilmington, DE).

### Pyrosequencing

The PyroMark Q24 BRAF and KRAS v2.0 assays (Qiagen) were used to detect mutations in BRAF (codon 600) and KRAS (codons 12, 13 and 61 in exons 2 and 3) according to the manufacturer’s recommendations. Novel pyrosequencing assays were developed for the analysis of known PIK3CA mutation hotspots in exon 9 (codons 542, 545, and 546) and exon 20 (codons 1043 and 1047). PCR primers and sequencing primers were designed using the PyroMark Assay Design 2.0 software (Qiagen). Forward (F) and reverse (R) PCR primers and sequencing primers (S) for PIK3CA were as follows (5’-3’): 9-F CAGCTCAAAGCAATTTCTACACG (biotin); 9-R CTCCATTTTAGCACTTACCTGTGAC; 9-S TG ACTCCATAGAAAATCTTT; 20-F GCAAGAGGCTTTGGAGTATTTC (biotin); 20-R AG ATCCAATCATTTTTGTTGTC; 20-S TTTTGTTGTCCAGCC. Briefly, ten nanogram of genomic DNA was used in 25 μl PCR reactions. Eight (PIK3CA) or 20 μl (BRAF and KRAS) of the PCR product was subsequently subjected to pyrosequencing using Streptavidin Sepharose High Performance (GE Healthcare, Uppsala, Sweden), PyroMark Gold Q96 reagents, PyroMark Q24 1.0.9 software, and a Q24 instrument (QIAGEN). All identified mutations were confirmed in a second analysis.

### MSI analysis

Determination of MSI status was performed using MSI Analysis System, version 1.2 (Promega, Madison, WI) with 6 ng genomic DNA and analysis of five mononucleotide repeat markers (BAT25, BAT26, NR-21, NR-24 and MONO-27). Analyses were performed on a 3130xl genetic analyzer (Applied Biosystems, Foster City, CA). According to guidelines from a National Cancer Institute workshop in 1997, samples were denoted MSI-High (MSI-H) if two or more of the five markers show instability, MSI-Low (MSI-L) if only one marker shows instability and microsatellite stable (MSS) if no markers display instability. In this study, MSI-L and MSS was grouped together in the interpretation of MSI data, therefore MSI refers to MSI-H and MSS refers to both MSS and MSI-L.

### SNP array analysis

Array experiments were performed according to the standard protocols for AffymetrixGeneChip® Mapping SNP 6.0 arrays (AffymetrixCytogenetics Copy Number Assay User Guide (P/N 702607 Rev2.), Affymetrix Inc., Santa Clara, CA). Briefly, 500 ng total genomicDNA was digested with a restriction enzyme (*Nsp, Sty*), ligated to an appropriate adapter for the enzyme, and subjected to PCR amplification using a single primer. After digestionwith DNase I, the PCR products were labeled with a biotinylatednucleotide analogue using terminal deoxynucleotidyltransferaseand hybridized to the microarray. Hybridized probes were captured by streptavidin-phycoerythrin conjugates using the Fluidics Station 450 and the arrays were finally scanned using the GeneChip® Scanner 3000 7G. Normalization and segmentation of genomic data was performed using BioDiscovery Nexus Copy Number 6.0 and the SNP Rank Segmentation algorithm [24,25] with default settings. Genome-wide average DNA copy number (ploidy) and the proportion of the genome with allelic imbalance were determined using Tumour Aberration Prediction Suite (TAPS) [15]. Average DNA copy number was calculated as the mean copy number of all genomic segments, weighted on segment length. Near diploid tumours were defined to have average copy number <2.5 and aneuploid tumours to have average copy number ≥2.5. SNP array data is available at GEO with accession number: (GSE62875).

### Statistical analyses

The Mann-Whitney U test was used in comparisons of non-parametric two group parameters, the Kruskal-Wallis test for multiple groups and the Chi-square test for dichotomous response parameters and to test differences in proportions between groups. A two-sided Fisher’s exact test was used instead of the Chi-square test when fewer than 30 cases where analysed in total or less than 10 cases in each group. Spearman’s rho was used to calculate the correlation coefficient (r). The odds ratio (OR) and the 95% confidence intervals (CI) were calculated according to Ahlbom et al. [26]. Differences were considered statistically significant if p < 0.05.

### Ethics

Ethical approval was obtained from the Ethics committee at Uppsala University, Uppsala, Sweden.

## Results

**Figure 1:**
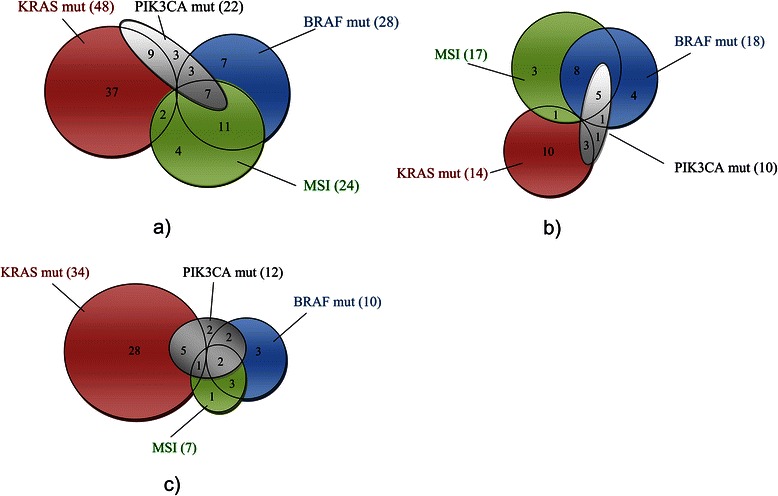
**Venn diagrams representing the interrelations of KRAS, BRAF, PIK3CA mutations and MSI in primary tumours from patients with colon cancer; a) the entire cohort (n = 121); b) non-disseminated disease (n = 53) and c) disseminated disease (n = 68).**

Of the 121 tumours analysed, 48 (40%) had KRAS mutations, the mutations where located in codon 12 (65%), codon 13 (31%) and codon 61 (4%). BRAF mutations were detected in 28 (23%) of the tumours and PIK3CA mutations were seen in 22 (18%) tumours mainly in exon 9 (n = 18; 82%) with 4 mutations in exon 20 (18%). MSI-H was detected in 24 (20%) tumours and MSI-L in 7 (6%). DNA copy number <2.5 were seen in 66 out of 116 (57%) tumours analysed. In Table 1 the main clinical and histopathological characteristics of the cohort are shown in relations to KRAS, BRAF and PIK3CA mutations and MSI and DNA copy number. The main findings were that KRAS mutation was associated with advanced disease stage, BRAF mutations were mainly found in right colon, PIK3CA was associated with poor tumour differentiation, MSI was more commonly seen in lower disease stage, larger and more poorly differentiated tumours. However, DNA copy number did not reveal any associations to the variables analysed (Table 1). The well-known mutual exclusiveness of KRAS and BRAF mutations was observed (Table 2 and Figure 1), and MSI was more prevalent in KRAS wild-type and BRAF mutated tumours (Table 2). PIK3CA mutations were in this cohort significantly associated with the presence of BRAF mutations and MSI (Table 2) and, in contrast to the mutual exclusive pattern of KRAS and BRAF mutations, PIK3CA mutations coexisted with mutations in the two other genes.

**Table 1:** **Clinical and histopathological relations of KRAS, BRAF and PIK3CA mutations and MSI (n = 121) and DNA copy number (n = 116) in primary tumours of patients with colon cancer**

	Total	Kras wt	Kras mut	p	Braf wt	Braf mut	p	PIK3CA wt	PIK3CA mut	p	MSS	MSI	p	DNA copy number		p
														<2.5	≥2.5	
***Number***	121	73	48		93	28		97	22		97	24		66	50	
***Age at diagnosis***																
Years (mean)	70	71	69	0,346	69	72	0,388	71	67	0,380	70	70	0,858	71	69	0,412
***Gender***																
Female	71	43	28	0.950	51	20	0.132	57	14	0.641	54	17	0.177	37	31	0.520
Male	50	30	20		42	8		42	8		43	7		29	19	
***Tumour location***																
Right colon	73	43	30	0.692	49	24	0.002	56	17	0.093	55	18	0.110	39	31	0.751
Left colon	48	30	18		44	4		43	5		42	6		27	19	
***Tumour stage***																
Stage II	40	29	11	0,008	28	12	0,160	35	5	0,147	27	13	0,010	26	14	0,247
Stage III	55	34	21		43	12		45	10		46	9		28	25	
Stage IV	26	10	16		22	4		19	7		24	2		12	11	
***Tumour size***																
<5 cm	38	22	16	0.749	31	7	0.639	31	7	0.100	36	2	0.011	21	16	0.972
≥5 cm	82	50	32		62	20		67	15		61	21		44	34	
Missing data	1															
***Differentiation***																
Poor	28	20	8	0,193	18	10	0.072	18	10	0.006	14	14	<0.001	18	9	0.274
Well-moderate	93	53	40		75	18		81	12		83	10		48	41	
***Mucinous***																
No	102	62	40	0,804	81	21	0.143	84	18	0.748	84	18	0.208	55	42	0.100
Yes	19	11	8		12	7		15	4		13	6		11	8	
***Perineural invasion***																
No	117	71	2	0.649	89	28	0.572	97	20	0.151	93	24	0.583	64	49	1.000
Yes	4	46	2		4	0		2	2		4	0		2	1	
***Vascular invasion***																
No	104	65	39	0,287	78	26	0.354	85	18	1.000	81	23	0.189	58	42	0.549
Yes	17	8	9		15	2		14	3		16	1		8	8	

Wt: wildtype; mut: mutation.

**Table 2:** **Correlations between KRAS, BRAF and PIK3CA mutations, MSI (n = 121) and DNA copy number (n = 115) in primary tumours from patients with colon cancer**

	BRAF				PIK3CA				MSI vs MSS				DNA copy number				
	Mut	Wt	p	r	Mut	Wt	p	r	MSI	MSS	p	r	<2.5	≥2.5	Missing	p	r
KRAS																	
Mutation	0	48	<0.001	-0.414	9	39	0.100	-0.003	2	46	<0.001	-0.338	22	22	3	0.169	-0.146
Wild type	28	45			13	60			22	51			46	25	3		
BRAF																	
Mutation					10	18	0.006	-0.213	18	10	<0.001	0.657	23	5		0.009	0.265
Wild type					12	81			6	87			45	42	6		
PIK3CA																	
Mutation									8	14	0.041	0.173	14	7	1	0.595	0.072
Wild type									16	83			54	40	5		
MSI																	
MSI													23	0	1	<0.001	0.416
MSS													45	47	5		

Wt: Wild type; Mut: Mutation; r: Correlation coefficient.

**Figure 2:**
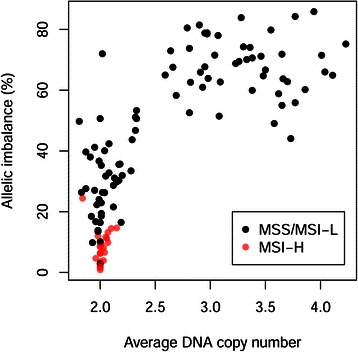
**MSS/MSI-L and MSI-H samples were plotted according to average DNA copy number and proportion of the genome with allelic imbalance (%).**

Tumours with average DNA copy number <2.5 frequently exhibited MSI and mutated BRAF. None of the tumours with MSI demonstrated an average DNA copy number ≥2.5 (Table 2 and Figure 2). On the contrary, 51 percent of the MSS tumours demonstrated an average DNA copy number ≥2.5, and were in all cases accompanied by a high proportion of the genome affected by allelic imbalance (Figure 2). However, average DNA copy number was neither associated with KRAS, nor PIK3CA mutation status (Table 2).

DNA copy number or PIK3CA mutations revealed no associations with disseminated disease or recurrence in the whole study cohort, or in various subgroup combinations of the cohort, and were therefore excluded from further analysis.

KRAS mutated tumours were more commonly seen in patients with disseminated disease. In contrast, BRAF mutations or MSI were less common in tumours from patients with disseminated disease or in those developing recurrence in disease stages II and III (Table 3). No statistically significant associations were seen when disease stages II and III were analysed separately (data not shown).

**Table 3:** **The associations of KRAS and BRAF mutations and MSI to the risk of recurrence and dissemination in patients with colon cancer**

	Disease stage II and III				Disseminated^¥^	Non disseminated^β^	Odds ratio (95% Confidence interval)	P
	Recurrence	No recurrence	Odds ratio (95% Confidence interval)	P				
n	42	53			68	53		
KRAS								
Mutation	18	14	2.09 (0.88-4.96)	0.092	34	14	2.75 (1.28-6.04)	0.009
Wild type	24	39			34	39		
BRAF								
Mutation	6	18	0.32 (0.16-0.91)	0.034	10	18	0.34 (0.14-0.81)	0.013
Wild type	36	35			58	35		
MSI								
MSI	5	17	0.29 (0.10-0.86)	0.027	7	17	0.24 (0.09-0.64)	0.005
MSS	37	36			61	36		
MSS only								
KRAS								
Mutation	18	13	0.95 (0.35-2.58)	0.279	33	13	2.08 (0.89-4.86)	0.087
Wild type	19	23			28	23		
BRAF								
Mutation	2	5	0.35 (0.06-1.96)	0.261	5	5	0.55 (0.15-2.06)	0.492
Wild type	35	31			56	31		
KRAS wild type only								
MSI								
MSI	5	16	0.38 (0.12-1.22)	0.168	6	16	0.31 (0.10-0.91)	0.041
MSS	19	23			28	23		
BRAF								
Mutation	6	18	0.39 (0.13-1.19)	0.115	10	18	0.49 (0.18-1.28)	0.142
Wild type	18	21			24	21		
BRAF wild type only								
MSI								
MSI	1	4	0.22 (0.02-2.09)	0.198	2	4	0.28 (0.04-1.60)	0.194
MSS	35	31			56	31		
KRAS								
Mutation	18	14	1.50 (0.59-3.84)	0.397	34	14	2.13 (0.90-4.99)	0.082
Wild type	18	21			24	21		
MSI and BRAF*								
BRAF wild type + MSS	35	31	3.55 (1.33-9.44)	0.013	56	31	3.31 (1.45-7.59)	0.004
BRAF mutation + MSS	2	5	0.48 (0.09-2.61)	0.459	5	5	0.76 (0.21-2.78)	0.747
BRAF mutation + MSI	4	13	0.32 (0.10-1.08)	0,050	5	13	0.24 (0.08-0.74)	0.011
BRAF wild type + MSI	1	4	0.30 (0.03-2.78)	0.379	2	4	0.37 (0.07-2.11)	0.403

^β^Non-disseminated: Disease stages II and III without recurrence; ^¥^Disseminated: Disease stages II and III with recurrence and stage IV.

*The comparison of each subgroup is made with all other groups.

Higher frequency of KRAS mutations was observed in tumours from patients with higher disease stages; 28% in stage II; 38% in stage III and 62% in stage IV. Whereas mutated BRAF, as well as MSI, were more frequent in lower disease stages; BRAF mutation frequency was 30% in stage II; 22% in stage III and 15% in stage IV and the frequency of MSI was: 33% in stage II; 16% in stage III and 8% in stage IV. When these genotypes were analysed separately in left and right colon, MSI and BRAF mutations were observed more frequently in the right colon and these molecular changes were present in both tumours from patients with, or without, recurrence in disease stages II and III and in disseminated disease (Table 4). For left colon, MSI and BRAF mutations could not be found in tumours from patients developing disease recurrence in stages II or III and were rare in those with disseminated disease (Table 4). On the contrary, KRAS mutations had a stronger association with disseminated disease in left compared with right colon (Table 4). Overall KRAS was the most frequently mutated gene in patients with disseminated disease (Figure 1c) and KRAS codon 12 glycine to valine mutations was seen in 10 of 34 KRAS mutated tumours in patients with disseminated disease compared to 2 of 14 KRAS mutated tumours in patients with non-disseminated disease (data not shown).

**Table 4:** **The prognostic associations of KRAS mutation, BRAF mutation and MSI in right versus left colon in 121 patients with colon cancer**

	Diseasestage II and III				All			
	Recurrence	No recurrence	Odds ratio (95% Confidence interval)	P	Disseminated^¥^	Non-disseminated^β^	Odds ratio (95% Confidence interval)	P
Rightcolon								
MSI	5	12	0,40 (0,12-1,31)	0,158	6	12	0,31 (0,10-0,95)	0.055
MSS	22	21			34	21		
Leftcolon								
MSI	0	5	*	0,057	1	5	0,11 (0,01-1,04)	0.069
MSS	15	15			27	15		
Rightcolon								
BRAFmutation	6	14	0,38 (0,12-1,21)	0,168	10	14	0,45 (0,17-1,22)	0.138
BRAFwildtype	21	19			30	19		
Leftcolon								
BRAFmutation	0	4	*	0,119	0	4		0.025
BRAFwildtype	15	16			28	16	*	
Rightcolon								
BRAF/MSIpresent	7	16	0,37 (0,12-1,12)	0,110	11	16	0,40 (0,15-1,07)	0.089
BRAF/MSI absent	20	17			29	17		
Leftcolon								
BRAF/MSIpresent	0	6	*	0,024	1	6	0,09 (0,01-0,79)	0.015
BRAF/MSI absent	15	14			27	14		
Rightcolon								
KRASmutation	13	10	2,13 (0,74-6,16)	0,157	20	10	2,3 (0,87-6,05)	0.089
KRASwildtype	14	23			20	23		
Leftcolon								
KRASmutation	5	4	2,00 (0,43-9,27)	0,451	14	4	4,00 (1,07-15,01)	0.041
KRASwildtype	10	16			14	16		

^β^Non-disseminated: Disease stage II and III without recurrence; ^¥^Disseminated: Disease stage II and III with recurrence and stage IV. *Not able to calculate OR because of 0 in one grupp.

In Table 3, patients with MSS tumours only, KRAS wild type only and BRAF wild type only are also presented according to molecular status, dissemination and recurrence. Among these subgroups, patients with KRAS wild type tumours that are MSI are less likely (p = 0.041) to have disseminated disease. Patients with KRAS mutated MSS tumours appear more likely to have disseminated disease, but recurrences in stages II and III disease were not more frequent when MSS tumours were KRAS mutated. The same trend for dissemination can be seen for BRAF wild type tumours with a KRAS mutation (Table 3). The OR for dissemination for BRAF mutated tumours is low both in MSS tumours and in KRAS wild type tumours; however these results are statistically non-significant.

In an attempt to identify specific subgroups of molecular markers that could help to detect patients with high or low risk of disease dissemination, or recurrence in stages II and III, several combinations of markers were of interest. Patients with tumours presenting both KRAS wild type and MSI had a reduced risk of dissemination (OR 0.22; 95% CI 0.08-0.62) and recurrence in disease stages II and III (OR 0.31; 95% CI 0.10-0.94) compared with all other groups. On the other hand, patients with tumours harbouring both BRAF wild type and MSS presented a higher risk of disseminated disease, and disease recurrence in stages II and III compared with all other groups (Table 3). Tumours with both BRAF mutation and MSI had the lowest risk for dissemination also marginally significant for lower risk for disease recurrence in stages II and III (Table 3). No statistically significant differences were seen when stages II and III were analysed separately with aforementioned subgroups (data not shown).

## Discussion

The present study revealed that tumour dissemination is less likely to occur in colon cancer patients with microsatellite instable (MSI) disease or mutated BRAF, as compared to patients with MSS or BRAF wild-type tumours. On the contrary, disseminated disease was more commonly observed in patients with mutated KRAS, as compared to their KRAS wild-type counterparts.

This study is among the first that describes frequencies of mutations and microsatellite instability in association with disease dissemination (metastatic disease either present at the time of diagnosis or developed during follow-up time) in a selected subset of colon cancer patients. The rationale behind including patients with stage II and III colon cancer, with and without recurrent metastatic disease, together with stage IV patients (metastatic disease at diagnosis), was to facilitate the detection of predictive genotypes in a cost-effective way. The applied unmatched case-control design enabled a smaller number of samples to be analysed, while the number of critical events was maintained. However, it should be noted that the reduced sample size of each subgroup, as a result of the applied selection criteria, also might limit the power to detect statistically significant differences between the subgroups. Furthermore, even based on a large material of over 600 frozen tissue samples, we were unable to include the planned number of stage II patients with metastatic recurrence. The strict quality requirements with regard to staging, surgery, and pathology contributed to this inability, but at the same time likely increased the validity of the results, as the influence of unrelated factors was minimised.

The observed mutation frequencies in the present investigation should be interpreted with caution, as this cohort is not population-based. Even so, the KRAS mutation frequency of 40% in this cohort was in good agreement with other published studies [27-29]. Moreover, we observed that the proportion of KRAS mutated patients increased with higher disease stage, a finding supported by Eklöf et al. [30], but not uniformly seen in other cohorts [31,32].Today KRAS mutation status is routinely analysed because of its predictive nature in patients receiving therapeutic antibodies against EGFR, with treatment restricted to patients with KRAS wild type tumours [33,34]. In addition to predictive power with regard to treatment response, the prognostic impact of mutated KRAS has been thoroughly studied in CRC. In the RASCAL II study, KRAS mutations were associated with worse prognosis compared to KRAS wild type in over 3000 patients with CRC, an association that was stronger in stage III than in stage II [31]. The association to worse prognosis was however restricted to KRAS 12Gly > Val in stage III disease [31,35]. In the present study, a similar trend of worse prognosis for KRAS 12Gly > Val mutated patients was observed. Additional studies have confirmed the association of KRAS mutations and poor prognosis [30,32,36-38]. Contrary to these results, two other prospective studies, including 1,404 and 315 patients respectively, did not demonstrate any major impact of KRAS mutations on prognosis [39,40].

In the present study, the BRAF mutation frequency (23%) was higher compared to the 5-17% previously reported in colorectal cancer [30,32,41], possibly explained by the fact that right-sided tumours were predominant in our cohort and BRAF mutations have been reported to mainly occur in tumours of the right colon [30,37,39-41]. BRAF mutations were associated with lower likelihood of tumour dissemination in the whole cohort, as well as lower likelihood of metastatic recurrence in a separate analysis of stage II and III tumours. This is in contrast to a majority of published studies, where BRAF mutations were mostly associated with worse prognosis [28,30,37,39,40,42,43] or did not exhibit a prognostic impact [30,38]. Of interest is that two recent studies showed that BRAF mutations were related to worse overall survival, but not to relapse-free survival [44,45], which may be explained by higher frequencies of BRAF mutations in older individuals [30,45].

BRAF and KRAS mutations were confirmed to be mutually exclusive in this study, as previously reported [46]. BRAF mutations were moreover significantly associated with MSI, also this in agreement with previous findings [37,47]. The good prognostic feature of patients with the MSI tumour type, also seen here, is well-established [38,48-50] and MSI has been reported to be prognostic in both stages II and III [48], stage II only [48,50] and stage III only [19]. As observed by others and similarly to BRAF mutations, MSI tumours were found to have larger tumour size, association with lower disease stage and poor differentiation. However, the frequently seen associations of MSI with right colon, mucinous tumour type and female gender was not seen in the present cohort possibly reflecting the differences in selection of patients compared with consecutive cohorts. Interestingly, of the patients with left-sided MSI tumours in the present cohort none developed recurrence. It is tempting to omit MSI analysis in left-sided colon cancers, as only about 5% of left-sided tumours are expected to be MSI, however this study indicates that MSI analysis can assist when selecting patients for adjuvant treatment even for left sided tumours. We were unable to find any publications that analysed the prognostic impact of MSI in left-sided colon cancers, as most studies state that the case number is too low for meaningful investigations of this subset [38].

MSI tumours are characterised by a defective DNA mismatch repairsystem and the consequential accumulation of mutations in tumour suppressor genes and oncogenes. Tumours that are MSS commonly exhibit another type of instability, CIN, with abundant large-scale genomic alterations that often lead to a higher average DNA copy number. In contrast to MSI, average DNA copy number is not routinely assessed. Therefore, in the present study, average DNA copy number was determined based on genome-wide SNP array analysis. A low average DNA copy number was associated with the presence of BRAF mutation and MSI, but no association with tumour dissemination nor disease recurrence was found, suggesting that the analysis of average DNA copy number would not improve routine diagnostics.

In addition to KRAS and BRAF mutations, it has been put forward that mutations in PIK3CA, the p110α catalytic subunit of phosphatidylinositol-4,5-bisphosphonate 3-kinase (PI3K) and a main player in the PI3K/AKT/mTOR pathway, might be of clinical relevance. Coexistence of PIK3CA exon 9 and 20 mutations has, mainly by one group, revealed worse prognosis in CRC [22,51]. The present study revealed that PIK3CA mutations were more common in MSI and BRAF mutated tumours. However, no significant association with tumour dissemination was observed, an observation supported by others [30].

Molecular analysis methods to detect the presence of mutations and chromosomal or microsatellite instability are unlikely to replace conventional pathological analysis, but can potentially help oncologists decide whether or not colon cancer patients should receive chemotherapy as an adjuvant treatment to reduce the risk of metastatic recurrence.

## Conclusions

The present study revealed that tumour dissemination is less likely to occur in colon cancer patients displaying MSI or BRAF mutation, whereas the presence of a KRAS mutation increases the likelihood of disseminated disease.

## Additional file

Additional file 1: Table S1. Clinical and histopathological data of the study cohort including 121 cases with primary colon cancer.
