# Supplementary material for: Microsatellite instability and mutations in BRAF and KRAS are significant predictors of disseminated disease in colon cancer
Source: BMC Cancer. 2015 Mar 14;15:125. doi: 10.1186/s12885-015-1144-x (PMC4364587; doi:10.1186/s12885-015-1144-x)
Supplement: Additional file 1: Table S1. — Clinical and histopathological data of the study cohort including 121 cases with primary colon cancer. [file 12885_2015_1144_MOESM1_ESM.docx]

Supplementary Table 1. Clinical and histopathological data of the study cohort including 121 cases with primary colon cancer.

|  | Total  *n*= | Stage II | | | | Stage III | | | | Stage IV | Disseminated^¥^ | Non-disseminated^β^ | *p* |
| --- | --- | --- | --- | --- | --- | --- | --- | --- | --- | --- | --- | --- | --- |
|  |  | Total | Recurrence | No recurrence | *p** | Total | Recurrence | No recurrence | *p*** |  |  |  |  |
|  | 121 | 40 | 15 | 25 |  | 55 | 27 | 28 |  | 26 | 68 | 53 |  |
| **Age at diagnosis** | 73 | 72 | 73 | 72 | 0.514 | 76 | 77 | 73 | 0.080 | 66 | 72 | 74 | 0.775 |
| **Gender** |  |  |  |  |  |  |  |  |  |  |  |  |  |
| Female | 71 | 22 | 8 | 14 | 0.870 | 32 | 17 | 15 | 0.480 | 19 | 42 | 29 | 0.435 |
| Male | 50 | 18 | 7 | 11 |  | 23 | 10 | 13 |  | 9 | 26 | 24 |  |
| **Tumor size** |  |  |  |  |  |  |  |  |  |  |  |  |  |
| <5 cm | 38 | 11 | 7 | 4 | 0.069 | 19 | 11 | 8 | 0.343 | 8 | 26 | 12 | 0.077 |
| ≥5 cm | 82 | 28 | 8 | 20 |  | 36 | 16 | 20 |  | 18 | 42 | 40 |  |
| Missing data | 1 | 1 | 0 | 1 |  | 0 | 0 | 0 |  | 0 | 0 | 1 |  |
| **Tumor location** |  |  |  |  |  |  |  |  |  |  |  |  |  |
| Right colon | 73 | 26 | 11 | 15 | 0.392 | 34 | 16 | 18 | 0.701 | 13 | 40 | 33 | 0.701 |
| Left colon | 48 | 14 | 4 | 10 |  | 21 | 11 | 10 |  | 13 | 28 | 20 |  |
| **Differentiation** |  |  |  |  |  |  |  |  |  |  |  |  |  |
| Poor | 28 | 5 | 1 | 4 | 0.633 | 16 | 9 | 7 | 0.496 | 7 | 17 | 11 | 0.302 |
| Well-moderate | 93 | 35 | 14 | 21 |  | 39 | 18 | 21 |  | 19 | 51 | 42 |  |
| **Mucinous** |  |  |  |  |  |  |  |  |  |  |  |  |  |
| No | 102 | 34 | 13 | 21 | 1.000 | 45 | 24 | 21 | 0.296 | 23 | 60 | 42 | 0.177 |
| Yes | 19 | 6 | 2 | 4 |  | 10 | 3 | 7 |  | 3 | 8 | 11 |  |
| **Neural invasion** |  |  |  |  |  |  |  |  |  |  |  |  |  |
| No | 117 | 39 | 14 | 25 | 0.375 | 52 | 24 | 28 | 0.112 | 26 | 64 | 53 | 0.130 |
| Yes | 4 | 1 | 1 | 0 |  | 3 | 3 | 0 |  | 0 | 4 | 0 |  |
| **Vascular invasion** |  |  |  |  |  |  |  |  |  |  |  |  |  |
| No | 104 | 38 | 14 | 24 | 1.000 | 45 | 22 | 23 | 1.000 | 21 | 57 | 47 | 0.446 |
| Yes | 17 | 2 | 1 | 1 |  | 10 | 5 | 5 |  | 5 | 11 | 6 |  |
| **Chemotherapy** |  |  |  |  |  |  |  |  |  |  |  |  |  |
| No | 67 | 36 | 13 | 23 | 0.484 | 24 | 16 | 7 | 0.014 | 8 | 37 | 30 | 0.806 |
| Yes | 54 | 4 | 2 | 2 |  | 29 | 11 | 21 |  | 18 | 31 | 23 |  |
| **Type of dissemination** |  |  |  |  |  |  |  |  |  |  |  |  |  |
| Liver metastasis |  |  | 11 |  |  |  | 20 |  |  | 16 | 47 |  |  |
| Lung metastasis |  |  | 5 |  |  |  | 10 |  |  | 2 | 17 |  |  |
| Other^#^ |  |  | 3 |  |  |  | 4 |  |  | 9 | 16 |  |  |

*Comparison of stage II patients with and without recurrence; **Comparison of stage III patients with and without recurrence; ^β^Non-disseminated: Disease stage II and III without recurrence; ^¥^Disseminated: Disease stage II and III with recurrence and stage IV; ^#^Carcinomatosis, paraaortal lymphnodes, cerebral.
